# Selective distillation phenomenon in two-species Bose-Einstein condensates in open boundary optical lattices

**DOI:** 10.1038/srep17101

**Published:** 2015-11-24

**Authors:** Xiao-Dong Bai, Mei Zhang, Jun Xiong, Guo-Jian Yang, Fu-Guo Deng

**Affiliations:** 1 Department of Physics, Applied Optics Beijing Area Major Laboratory, Beijing Normal University, Beijing 100875, China

## Abstract

We investigate the formation of discrete breathers (DBs) and the dynamics of the mixture of two-species Bose-Einstein condensates (BECs) in open boundary optical lattices using the discrete nonlinear Schrödinger equations. The results show that the coupling of intra- and interspecies interaction can lead to the existence of pure single-species DBs and symbiotic DBs (i.e., two-species DBs). Furthermore, we find that there is a selective distillation phenomenon in the dynamics of the mixture of two-species BECs. One can selectively distil one species from the mixture of two-species BECs and can even control dominant species fraction by adjusting the intra- and interspecies interaction in optical lattices. Our selective distillation mechanism may find potential application in quantum information storage and quantum information processing based on multi-species atoms.

Bose-Einstein condensates (BECs) trapped in periodic optical potentials are an invaluable tool to study fundamental and applied aspects of quantum optics, quantum computing, and solid state physics^1^,^2^,^3^,^4^. It is important to understand the dynamics and transport properties of BECs in optical lattices. One of the most interesting features of BECs in nonlinear lattices is the existence of localized excitation, which can propagate without changing its shape as a result of the balance between nonlinearity and dispersion^5^,^6^,^7^,^8^. This phenomenon is also referred to the formation of discrete breathers (DBs). DB is an interesting discovery in nonlinear science and has been observed in other physical systems as well, such as micromechanical cantilever arrays^9^, antiferromagnet systems^10^,^11^, Josephson-junction arrays^12^,^13^, nonlinear waveguide arrays^14^,^15^, Tonks gas^16^, and some dissipative systems^17^. In single-species BECs, many properties of DBs have been investigated theoretically and experimentally in the last decade^18^,^19^,^20^,^21^,^22^,^23^,^24^,^25^,^26^. One of its interesting properties is that the DBs are attractors and can slow down the relaxation processes in dissipative systems^27^,^28^,^29^. Moreover, some studies^29^,^30^,^31^ on the collision of a stationary DB with a lattice excitation (a moving breather or phonon) show that if the amplitude of the lattice excitation is small, it will be reflected entirely from the DB, while with the amplitude beyond a specific threshold, a part of the incident atoms transmit through the DB.

The previous works are mainly focused on single-species BECs. Actually, the two- and multi-species BECs have been observed in experiment and attracted much attention. In 2008, Thalhammer *et al*.^32^ observed an interesting mixture of heteronuclear BECs in experiment, where ^41^K and ^87^Rb atoms are condensed together in an optical lattice. An important property of this mixture is that the interspecies scattering length describing the effective colliding interaction between ^41^K and ^87^Rb atoms can be tuned over a wide (both positive and negative) range using a magnetic Feshbach resonance, and their own intraspecies scattering length remains positive for each species. That is, in this mixture both the inter- and intraspecies interactions can be varied and controlled completely. Subsequently, both the stable mixture^33^ of the isotopes ^168^Yb and ^174^Yb, and the unstable mixture^34^ of the isotopes ^174^Yb and ^176^Yb were obtained. In 2011, two-species BECs have been realized^35^ with the mixture of two hyperfine states of ^87^Rb, which is spin-orbit-coupled (SO-coupled) BECs.

Recently, some interesting physical phenomena and unique properties have been discovered in multi-species BECs in optical lattices, such as multi-species gap solitons in spinor BECs^36^, dark-dark solitons and modulational instability in miscible two-species BECs^37^, unstaggered-staggered solitons^38^, and the other two-species solitons^39^,^40^,^41^,^42^,^43^,^44^,^45^,^46^ in two-species BECs. Also, it has been found that the mixing with the second atomic species can lead to some different physical phenomena^47^,^48^. For example, the interspecies interaction of the two-species BECs can result in the phase separation in a harmonic trap, i.e., the two species may be immiscible^49^,^50^. In 2008, Papp *et al*.^51^ found that the repulsive interaction between atoms of different species can leave the mixture of two-species BECs far from its ground state in experiment.

In this paper, we numerically investigate the formation of DBs in two-species BECs inside open optical lattices using the discrete nonlinear Schrödinger equations (DNLSEs). We find that the coupling of intra- and interspecies interaction can lead to the existence of pure single-species DB and symbiotic DBs, i.e., the DBs of species 1 and 2 locate together with the same or different species fraction at the same sites in open optical lattices. Furthermore, we explore the dynamics of the mixture in two-species BECs with a pure single-species DB in open optical lattices. Interestingly, we find that there is a selective distillation phenomenon in both the mixture of initial condition selected randomly and that of symbiotic DB. That is, by adjusting the interspecies interaction one can make one species transmit through the DB and the other be blocked, therefore increasing the relative proportion of the ultracold atoms in the former. Moreover, one can also improve the dominant specie fraction of the mixture of two-species BECs by tuning the interspecies interaction in three mixtures: initial condition selected randomly, moving symbiotic DBs, and stable symbiotic DBs. This phenomenon is potentially useful in quantum information storage and quantum information processing based on multi-species atoms.

## Results

### The model of two-species Bose-Einstein condensates

Two-species BECs can be created by simultaneously confining different atomic species in the same magnetic trap, including those of two different kinds of atoms and those of the same atoms in two different hyperfine states. For instance, a strongly repelling two-species system of different species can be created using ^41^K-^87^Rb atoms in an optical lattice^32^. Another two-species BECs were experimentally realized in hyperfine spin states of ^87^Rb, 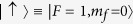 and 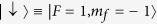, which is called SO-coupled BECs and resulted from a pair of counterpropagating Raman beams coupling the atomic states^35^ 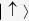 and 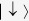. In these systems, the inter- and intraspecies interaction strengths can be controlled by a magnetic Feshbach resonance or adjusting the angle between the Raman beams.

We start our study with the following coupled Gross-Pitaevskii equations (GPEs) describing the dynamics of the two-species BECs^52^,^53^,^54^


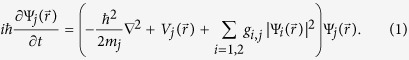


Here 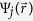 denotes the condensate for species *j* (= 1, 2). The coefficient *g*_*i*,*j*_ represents the interaction between two atoms from species *i* and *j*, which is defined as


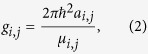


where *a*_*i*,*j*_ is the intraspecies (*i* = *j*) or interspecies (*i* ≠ *j*) scattering lengths and *μ*_*i*,*j*_ = *m*_*i*_*m*_*j*_/(*m*_*i*_ + *m*_*j*_) is the reduced mass of the atomic pair. The external potential 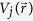 is generally a superposition of a harmonic trapping potential 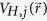 and the periodic optical lattice potential 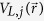, that is,


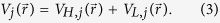


Here


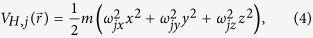



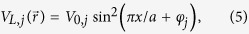


where *a* is the lattice spacing. When the lattices are sufficiently deep, one can work in the tight-binding limit, and the condensate is well localized around potential minima. The condensate parameter can be written as


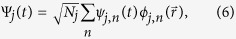


where *n* ( = 1, …, *M*) is the index of the site and 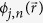 accounts for the ground state of the correspondingly isolated *n*-th potential. *M* is the number of lattice sites. |*ψ*_*j*,*n*_(*t*)|^2^ represents the number of the *j*-th species atoms at the *n*-th lattice site. By inserting Eq. (6) into Eq. (1) and integrating out the spatial degree of freedom, one can obtain DNLSEs^55^,^56^:


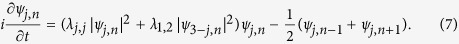


The total atomic population inside the optical lattice for each of the species 1 and 2 is 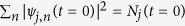. Here the atomic distribution of each species over the entire lattice is normalized to unity: *N*_*j*_(*t* = 0) = 1. The wave functions can be assumed as *ψ*_*j*,*n*_ = *A*_*j*,*n*_ exp(*iθ*_*j*,*n*_), where *A*_*j*,*n*_ and *θ*_*j*,*n*_ represent the amplitudes and the phases of species *j* at site *n*, respectively. In Eq. (7), the time has been re-scaled *t* → [*ħ*/(2*J*)]*t* with the assumption 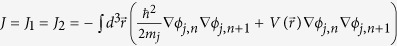 being the tunneling rate between the nearest-neighbor sites. The intra- and interspecies interactions are described by the parameters 
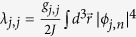
 and 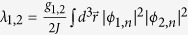, respectively. Here, we focus on the repulsive cases, i.e., *λ*_*j*,*j*_ > 0 and *λ*_1,2_ > 0.

### Formation of discrete breathers in two-species Bose-Einstein condensates

The formation of DBs in two-species BECs (*N*_1_ = *N*_2_) in open optical lattices can be investigated systematically by introducing the initial effective mean-field intra- and interspecies interactions per site as^28^,^29^


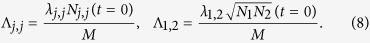


It has been demonstrated that in single-species BECs if the initial effective mean-field interaction Λ is larger than a critical value Λ^*^ ≈ 0.472 (which is gained from Eq. (17) of ref. 28 when *M* = 81), because of self-localization mechanism, the stable DBs can be created in open optical lattices with boundary dissipation^28^,^29^. If Λ is less than Λ^*^, DBs cannot be formed and atoms will decay. In two-species BECs, the DBs are still rooted from self-localization mechanism. If Λ_1,2_ = 0, species 1 and 2 are independent and one can get the corresponding critical values 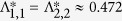. If Λ_1,2_ ≠ 0, species 1 and 2 are dependent, and for the special case Λ_1,1_ = Λ_2,2_ = 0, one can analogize the BECs with species 1 and 2 to single-species BECs and its corresponding critical value is 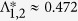. Here and after, we assume the critical value is Λ_*b*_ = 0.472. According to the difference of the parameters Λ_1,1_ and Λ_2,2_, the following investigation should be divided into three cases: (I) Λ_1,1_ = Λ_2,2_ = 0.1 < Λ_*b*_; (II) Λ_1,1_ = Λ_2,2_ = 0.6 > Λ_*b*_; (III) Λ_1,1_ = 0.1 < Λ_*b*_, and Λ_2,2_ = 0.6 > Λ_*b*_. In these three cases, the interspecies interaction Λ_1,2_ will paly an important role and will impact strongly on the formation and dynamics of DBs for two-species BECs in open optical lattices.

Let us consider the dissipation case with atoms initially distributed uniformly at each site with random phases *θ*_*j*,*n*_, which reads


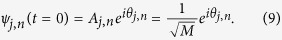


Here *θ*_*j*,*n*_ ∈ [0, 2*π*] is an arbitrary value. We assume that the boundary dissipation rates of lattices at sites 1 and M are *γ*_1_ = *γ*_2_ = 0.3 for both species.

**Figure 1:**
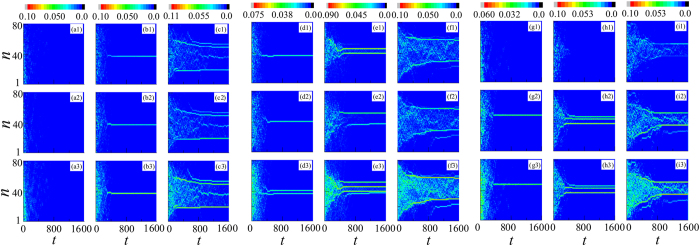
Formation of DBs of two-species BECs in open optical lattices with *M* = 81 sites. The color code shows |*ψ*_1,*n*_|^2^ (normalized to 1 at *t* = 0 for species 1), |*ψ*_2,*n*_|^2^ (normalized to 1 at *t* = 0 for species 2), and |*ψ*_1,*n*_|^2^ + |*ψ*_2,*n*_|^2^ in the first, the second, and the third rows, respectively. The initial condition is a homogeneously populated lattice with random phases at each site randomly drawn from [0, 2*π*]. The boundary dissipation rates at sites 1 and *M* are *γ*_1_ = *γ*_2_ = 0.3. The other parameters are chosen as follows: Λ_1,2_ = 0.1 < Λ_*b*_ for (a1–a3), (d1–d3), and (g1–g3); Λ_1,2_ = 0.5 > Λ_*b*_ for (b1–b3), (e1–e3), and (h1–h3), and Λ_1,2_ = 1 > Λ_*b*_ for (c1–c3), (f1–f3), and (i1–i3). (**a**–**c**) Λ_1,1_ = Λ_2,2_ = 0.1; (**d**–**f**) Λ_1,1_ = Λ_2,2_ = 0.6; (**g**–**i**) Λ_1,1_ = 0.1 and Λ_2,2_ = 0.6.

The formation of DB in two-species BECs in open optical lattices with *M* = 81 for the three cases (I), (II), and (III) with different Λ_1,2_ are shown in Fig. 1. In the first row, the color code shows |*ψ*_1,*n*_|^2^, which is normalized to 1 at *t* = 0 and describes the density of species 1. In the second and the third rows, the color codes show |*ψ*_2,*n*_|^2^ which is also normalized to 1 at *t* = 0 and describes the density of species 2, and |*ψ*_1,*n*_|^2^ + |*ψ*_2,*n*_|^2^ describing the sum density of both species 1 and 2, respectively.

In Fig. 1(a1–a3), Λ_1,1_ = Λ_2,2_ = Λ_1,2_ = 0.1 < Λ_*b*_ is very small, one can see that there is no DB in both species 1 and 2, and the atoms in the lattices get dissipated. That is, when both intra- and interspecies interactions are smaller than their critical values, no DBs can be formed. In Fig. 1(b1–b3), Λ_1,2_ = 0.5 is a bit larger than the critical value Λ_*b*_, and one DB can be formed for each of species 1 and 2 and they are located in the same position, as shown in Fig. 1(b3). It can be called a symbiotic DB. In Fig. 1(c1–c3) with Λ_1,2_ = 1 > Λ_*b*_, a few DBs can be formed for each of species 1 and 2, and the locations of DBs for species 1 are the same as those for species 2. That is, if Λ_1,2_ > Λ_*b*_ and Λ_1,1_ = Λ_2,2_ < Λ_*b*_, the properties of the two-species BECs are determined nearly by interspecies interactions Λ_1,2_, not the intraspecies interactions Λ_1,1_ and Λ_2,2_. Under this condition, the mixture of species 1 and 2 are analogous to single-species, and hence its properties are similar to those of single-species BECs. Thus, one can see that the dynamical properties of species 1 and 2 are exactly the same, and the DBs of the two species always co-exist in the same positions, as shown in Fig. 1(b2–c3).

In Fig. 1(d1–d3), Λ_1,1_ = Λ_2,2_ = 0.6 > Λ_*b*_ and Λ_1,2_ = 0.1 < Λ_*b*_. It is seen that the properties of the species 1 and 2 are determined by their intraspecies interactions Λ_1,1_ and Λ_2,2_, but not interspecies interactions Λ_1,2_. At this time, species 1 and 2 can be considered as two non-interacting species. Thus, one can see that one DB is formed in each of species 1 and 2. Different from those in Fig. 1(b1–b3), the two DBs of species 1 and 2 are not located at the same positions, as shown in Fig. 1(d3). In Fig. 1(e1–e3), Λ_1,2_ = 0.5 is larger a little than the critical value Λ_*b*_, and two DBs can be formed in each of species 1 and 2 and they are located at the different positions, as shown in 0.5(e3). That is, the formation process of DBs in species 1 and 2 are still independent. In both Fig. 1(f1–f3) Λ_1,2_ = 1 > Λ_*b*_, species 1 and 2 cannot be considered as the two independent species, and two or more strong DBs can be formed for each of species 1 and 2 and they co-exist in the same positions. Moreover, when Λ_1,2_ is large enough, the DB composed of only species 1 or 2 can prevent atoms of both species from dissipating out of the lattices, as shown in Fig. 1(f1–f3).

In Fig. 1(g1–g3) (Λ_1,2_ = 0.1) and (h1–h3) (Λ_1,2_ = 0.5), the formation processes of DBs in species 1 are determined by the interspecies interactions Λ_1,2_, while those in species 2 are determined by both the interspecies interactions Λ_1,2_ and intraspecies interactions Λ_2,2_. Thus, one can see that no DB is formed in species 1, as shown in Fig. 1(g1, and one or more DBs can be formed in species 2, as shown in Fig. 1(g2. In Fig. 1(i1–i3) with Λ_1,2_ = 1 > Λ_*b*_, due to the interplay of species 2, DBs can be formed not only in species 2 but also in species 1. The DBs of species 1 are weaker than those of species 2. That is, the formation of DBs of the two-species BECs is dominated by species 2, and the DBs of species 1 are like appurtenances.

## Selective Distillation of Ultracold Atomic Gas

From Fig. 1(f1–f3, one can see that the DBs of both species 1 and 2 can prevent the atoms from transferring through them when the interspecies interactions Λ_1,2_ are large enough. However, Fig. 1(a1–a3) predicts that species 1 and 2 are independent of each other when the interspecies interaction Λ_1,2_ is small or even vanish, that is, the DB of species *j* has an important effect on the transfer process of its own but not on the other species 3 − *j*. With this interesting mechanism, we propose a theoretical scheme to selectively distill one species from the mixture of two-species BECs in optical lattices. In order to describe the principle of our scheme clearly, let us define two new parameters,


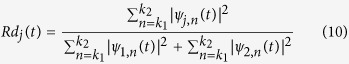


and


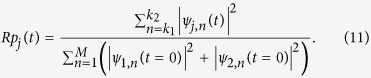


*Rd*_*j*_(*t*) describes the dominant species fraction of species *j* in the mixture of two-species BECs from the sites *k*_1_ to *k*_2_ in the optical lattice at time *t*, while *Rp*_*j*_(*t*) describes the relative proportion of the atoms in species *j* from the sites *k*_1_ to *k*_2_ in the mixture to all the atoms in the entire optical lattice. Here our investigation is mainly focused on two typical cases, i.e., initial condition selected randomly and moving symbiotic DB. It is worth noticing that we focus on the impact of DB on the dynamics of BECs, where the DB mainly occupies three sites and the effective interaction is Λ_*i*,*j*_ = *λ*_*i*,*j*_/3. For convenience, we choose *λ*_*i*,*j*_ to describe the dynamics of two-species BECs below, and this parameter has been used in previous works^31^,^57^.

### Selective distillation phenomenon in the chaos mixture of BECs

Let us assume that the initial condition is chaos with random amplitudes and phases at sites 1 to 20 in optical lattices with *M* = 81, and the other sites are empty (that is, their amplitudes are zero). There is a pure DB of species 2 at the middle site of optical lattice. That is, the initial condition reads as


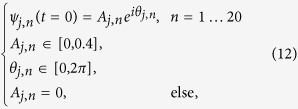



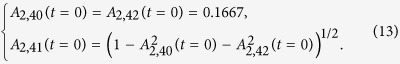


Eq. (12) represents the initial condition selected randomly, and *A*_*j*,*n*_ and *θ*_*j*,*n*_ are arbitrary values. Eq. (13) represents the DB of species 2.

**Figure 2:**
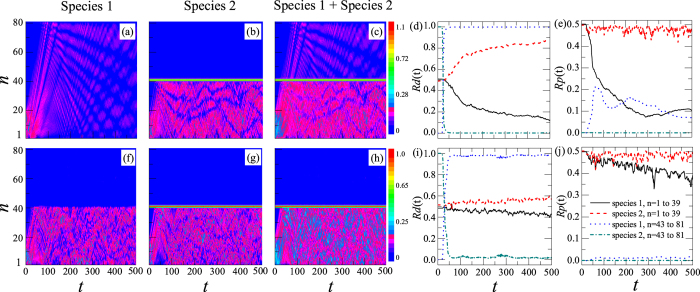
Selective distillation phenomenon in the chaos mixture of two-species BECs. The initial conditions are presented in Eqs (12) and (13). The color codes show |*ψ*_1,*n*_|^2^, |*ψ*_2,*n*_|^2^, and |*ψ*_1,*n*_|^2^ + |*ψ*_2,*n*_|^2^ in the first, the second, and the third columns, respectively. In the fourth and fifth columns, the solid and dashed lines represent the *Rd*_*j*_(*t*) and the *Rp*_*j*_(*t*) of species 1 and 2 at sites ranging from 1 to 39, respectively. The dotted and dot-dashed lines represent the *Rd*_*j*_(*t*) and the relative proportions of species 1 and 2 at sites ranging from 43 to 81, respectively. They are obtained from Eqs (10) and (11). In all cases, *λ*_1,1_ = *λ*_2,2_ = 4, *λ*_1,2_ = 0 in (**a**–**e**), and *λ*_1,2_ = 3 in (**f**–**j**).

Using the initial condition and Eqs (8) and (9) with *γ*_1_ = *γ*_2_ = 0, we simulate numerically the dynamics of two-species BECs, shown in Fig. 2 with *λ*_1,1_ = *λ*_2,2_ = 4. The color codes show |*ψ*_1,*n*_|^2^, |*ψ*_2,*n*_|^2^, and |*ψ*_1,*n*_|^2^ + |*ψ*_2,*n*_|^2^ in the first, the second, and the third columns, respectively. In the fourth and fifth columns, the different lines represent the *Rd*_*j*_(*t*) and *Rp*_*j*_(*t*) for species 1 and 2 at the different sites ranging from 1 to 39 or from 43 to 81, respectively.

In Fig. 2(a–e), *λ*_1,2_ = 0, which means these two species are independent of each other. If the DB is composed of only species 2, it has effect only on the dynamics of species 2, but not on that of species 1. As shown in Fig. 2(a–c), the part composed of species 1 in chaos can transmit through the DB without any hindrance, but the part composed of species 2 in chaos has been blocked by the DB. The change of the *Rd*_*j*_(*t*) of the two species is shown in Fig. 2(d). When *t* = 0, *Rd*_*j*_(*t* = 0) at the sites 1–39 are 50%, shown with the solid and dashed lines, respectively. At sites 43–81, since a small number of atoms of the DB can move to this area, there is only species 2, no species 1, that is, the *Rd*_*j*_(*t*) of species 1 and 2 are 0 and 1, respectively, shown with the dotted and dash-dotted lines in Fig. 2(d) at the beginning (*t* ~ 0), respectively. Subsequently, the *Rd*_*j*_(*t*) change with time *t*. After 24 time steps, the chaos extends to the DB, and then a part of species 1 transmits through it. At sites 43–81, the *Rd*(*t*) of species 1 first increases and then approaches the stable value of nearly 100%, but that of species 2 is nearly 0. In other words, species 1 with a high *Rd*_1_(*t*) is extracted from the chaos at the sites 1–39 when *λ*_1,2_ is far smaller than the critical value. We call this phenomenon the distillation of ultracold atomic gas.

To show this distillation phenomenon explicitly, we calculate the *Rp*_*j*_(*t*) of species 1 and 2 at the two different areas (one is composed of the sites from 1 to 39 and the other from 43 to 81), shown in Fig. 2(e). When *t* = 0, *Rp*_*j*_(*t* = 0) of species 1 and 2 at sites 1–39 in the mixture are 50%, shown with the solid and dashed lines, respectively, and *Rp*_*j*_(*t*) at sites 43–81 are 0, shown with the dotted and dash-dotted lines, respectively. After 24 time steps, *Rp*_2_(*t*) at sites 1–39 and 43–81 are constant, and *Rp*_1_(*t*) at sites 1–39 decrease and that at sites 43–81 can increase to 21.6%. That is, at most 21.6% of species 1 can be distilled from the mixture, and *Rp*_1_(*t*) at sites 43–81 (the dotted line) can represent the efficiency of this distillation.

When *λ*_1,2_ = 3, one can see that both species 1 and 2 are prevented by the DB and cannot transmit through it, as shown in Fig. 2(f–h). In Fig. 2(i), the *Rd*_*j*_(*t*) of species 1 and 2 at sites 43–81 are also nearly 100% and 0 at last, respectively. However, *Rp*_1_(*t*) at sites 43–81 is about 1%, that is, the number of atoms that transmit through the DB is very small due to the blocking of the DB, and species 1 is not effectively distilled. Thus, the DB plays a role of inhibitting the transmission of both species 1 and 2 when *λ*_1,2_ is much larger than the critical value.

From the discussion above, one can see that the distillation for the chaos depends strongly on the species of DB and the value of *λ*_1,2_. The former decides which species will be distilled, and the latter can control the efficiency of distillation. Therefore, by adjusting the interspecies interaction *λ*_1,2_, one can make one species transmit through the DB and the other be blocked, and increase the dominant species fraction of the ultracold atoms in the former. We call this the selective distillation phenomenon for the ultracold atoms in the mixture of two-species BECs.

### Selective distillation phenomenon in the mixture of moving symbiotic DB

Let us assume that a symbiotic DB of two-species BECs moves to a pure DB composed of species 2 which is represented by Eq. (13). The initial condition for the moving symbiotic DB reads as


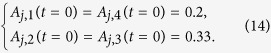


**Figure 3:**
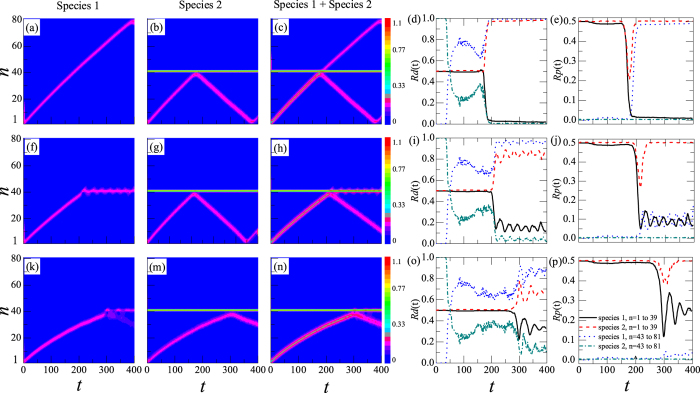
Selective distillation phenomenon of a moving symbiotic DB in two-species BECs. The initial conditions are presented in Eqs (13) and (14). The color codes show |*ψ*_1,*n*_|^2^, |*ψ*_2,*n*_|^2^, and |*ψ*_1,*n*_|^2^ + |*ψ*_2,*n*_|^2^ in the first, the second, and the third columns, respectively. In the fourth and fifth columns, the solid and dashed lines represent the *Rd*_*j*_(*t*) and *Rp*_*j*_(*t*) of species 1 and 2 at sites ranging from 1 to 39, respectively. The dotted and dot-dashed lines represent the *Rd*_*j*_(*t*) and *Rp*_*j*_(*t*) of species 1 and 2 at sites ranging from 43 to 81, respectively. They are obtained from Eqs (10) and (11). In all cases, *λ*_1,1_ = *λ*_2,2_ = 4, Λ_1,2_ = 0 in (**a**–**e**), *λ*_1,2_ = 0.2 in (**f**–**j**), and *λ*_1,2_ = 0.35 in (**k**–**p**).

The dynamics of the moving DB under DNLSE with three given interspecies interactions *λ*_1,2_ is shown in Fig. 3 with *γ*_1_ = *γ*_2_ = 0 and *λ*_1,1_ = *λ*_2,2_ = 4.

In Fig. 3(a–e), *λ*_1,2_ = 0. When the symbiotic DB moves to the stable DB, the part composed of species 1 of this moving DB can transmit through the stable DB without any hindrance, but that of species 2 is reflected by the stable DB, as shown in Fig. 3(a–c). The *Rd*_*j*_(*t*) and *Rp*_*j*_(*t*) of these two species are shown in Fig. 3(d,e), respectively. When *t* = 0, *Rd*_*j*_(*t*) and *Rp*_*j*_(*t*) of the two species in this symbiotic DB are nearly 50%, as shown in Fig. 3(d,e) with the solid and dashed lines, respectively. After 169 time steps, this symbiotic DB arrives at the stable DB and then collides with it. From Fig. 3(c), one can see that the transmitted part is composed of species 1. Accordingly, *Rd*(*t*) and *Rp*(*t*) of species 1 at sites 43–81 is increased to nearly 100% and 48.8%, shown in Fig. 3(d,e) with the dotted lines, respectively. That is, species 1 is distilled from the symbiotic DB with a high efficiency.

When *λ*_1,2_ = 0.2, the dynamics of the moving symbiotic DB is shown in Fig. 3(f–j). Different from Fig. 3(a–e), the part composed of species 1 of this symbiotic DB does not transmit through the stable DB but mixes with it, and the part composed of species 2 is reflected, as shown in Fig. 3(f–h). Their *Rd*_*j*_(*t*) and *Rp*_*j*_(*t*) are presented in Fig. 3(i,j), respectively. One can find that the *Rd*(*t*) of species 1 at sites 43–81 increases suddenly to a relatively stable value at time *t* = 200. During this time, *Rp*(*t*) of species 1 at sites 43–81 increases to 10%.

When *λ*_1,2_ = 0.35, the dynamics of the moving symbiotic DB is shown in Fig. 3(k–p). In the part composed of species 1 of this DB, some is mixed with the stable DB and the other part is reflected, as shown in Fig. 3(k), which is a new phenomenon and not yet understood so far. The part composed of species 2 of this moving DB is reflected by the stable DB. Their *Rd*_*j*_(*t*) and *Rp*_*j*_(*t*) are presented in Fig. 3(o,m), respectively. One can find that the *Rd*(*t*) of the species 1 at sites 43–81 is lower than that in Fig. 3(d–i), and *Rp*_1_(*t*) is 2%, shown with dotted lines in Fig. 3(m).

The dynamics of the symbiotic DB shows that there is also a selective distillation phenomenon in the transport for the moving atoms in two-species BECs with a DB. One can selectively distil one species from the moving symbiotic DB and control its *Rd*_*j*_(*t*) by adjusting the interspecies interaction *λ*_1,2_.

Certainly, there exist some atoms including species 1 and 2 escaping out of the symbiotic DB and spreading freely to the stable DB. Since the stable DB can prevent the atoms from species 2 but not 1, after 47 time steps, the *Rd*_*j*_(*t*) of species 1 and 2 change suddenly, shown in Fig. 3(d,i,o).

### Selective distillation for controlling the *Rd*_*j*_(*t*) of two-species BECs

It is interesting to control the *Rd*_*j*_(*t*) of two-species BECs in open optical lattices with a stable symbiotic DB by using selective distillation (here *γ*_1_ = *γ*_2_ = 0.3). As shown in the section of results before, when Λ_1,1_ = 0 and Λ_2,2_ > Λ_*b*_, species 1 will decay completely and the DB can be created in species 2 if Λ_1,2_ = 0. However, if Λ_1,2_ is large, these two species can be co-localized at the same location. That is, Λ_1,2_ plays an important role in the dynamics of the mixture, which means that one can control the *Rd*_*j*_(*t*) of the mixture of the two-species BECs by manipulating the corresponding interspecies interaction.

**Figure 4:**
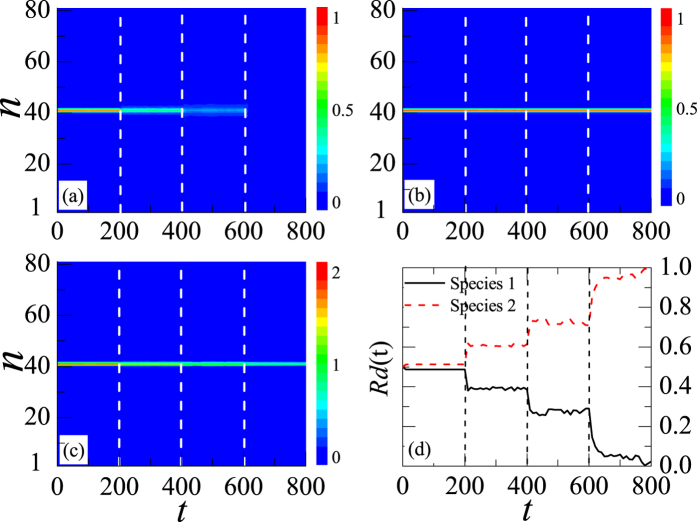
Selective distillation phenomenon of a stable symbiotic DB in two-species BECs. The color codes show |*ψ*_1,*n*_|^2^ (normalized to 1 at *t* = 0 for species 1), |*ψ*_2,*n*_|^2^ (normalized to 1 at *t* = 0 for species 1), and |*ψ*_1,*n*_|^2^ + |*ψ*_2,*n*_|^2^ in (**a**–**c**), respectively. The white dotted lines predict that, at the time *λ*_1,2_ starts to vary. (**d**) The changing of *Rd*_*j*_(*t*) for this two species with time. Its values refer to the symbiotic DB and are gained from Eq. (12), where *k*_1_ = 40 and *k*_2_ = 42. The solid and dotted lines represent the *Rd*_*j*_(*t*) of species 1 and 2, respectively. Here, *λ*_1,2_ = 3, 0.7, 0.3, and 0 in the ranges from the time steps 0–200, 200–400, 400–600 and 600–800, respectively. *λ*_1,1_ = 0 and *λ*_2,2_ = 9 at all time.

Let us assume that this mixture is initially a stable symbiotic DB, where the *Rd*_*j*_(*t*) of the two species are 50%, and this mixture locates at the middle site in optical lattices with *M* = 81. It can be described as


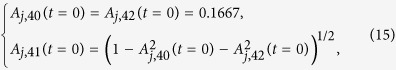


where *j* (=1, 2) represents the two different species. By varying *λ*_1,2_ every 200 time steps, the dynamics of the system is shown in Fig. 4. The white dotted lines label the time that *λ*_1,2_ starts to vary. Figure 4(d) describes the *Rd*_*j*_(*t*) change of species 1 and 2.

From Fig. 4(a), one can clearly find that when 0 < *t* < 200 and *λ*_1,2_ = 3, the density of species 1 is stable. The *Rd*_*j*_(*t*) of species 1 and 2 near to be 50%, as shown in the area from *t* = 0 to *t* = 200 in Fig. 4(d). From *t* = 200 to *t* = 400, *λ*_1,2_ turns to be 0.7. The density of species 1 decreases suddenly to another stable value. Accordingly, the *Rd*_*j*_(*t*) of species 1 (2) decreases (increases) suddenly to another stable value, as shown in the area from *t* = 200 to *t* = 400 in Fig. 4(d). Similarly, from *t* = 400 to *t* = 600, *λ*_1,2_ becomes much smaller to be 0.3. During this time, the density of species 1 continues to decrease, and the *Rd*_*j*_(*t*) of species 1 (2) decreases (increases) suddenly to a different stable value, as shown in the area from *t* = 400 to *t* = 600 in Fig. 4(d). At *t* = 600, *λ*_1,2_ decreases to zero, and species 1 will decay completely. Accordingly, the *Rd*_*j*_(*t*) of species 1 decreases to 0, and that of species 2 increases to 100%, as shown in the area from *t* = 600 to *t* = 800 in Fig. 4(d). In the entire process, species 2 does not change and is always localized, as shown in Fig. 4(b). The sum density of both species 1 and 2 has been presented in Fig. 4(c). It predicts that each *λ*_1,2_ corresponds to a specific *Rd*_*j*_(*t*) of this mixture. Consequently, the *Rd*_*j*_(*t*) of the two-species BECs mixture can be controlled by adjusting *λ*_1,2_.

**Figure 5:**
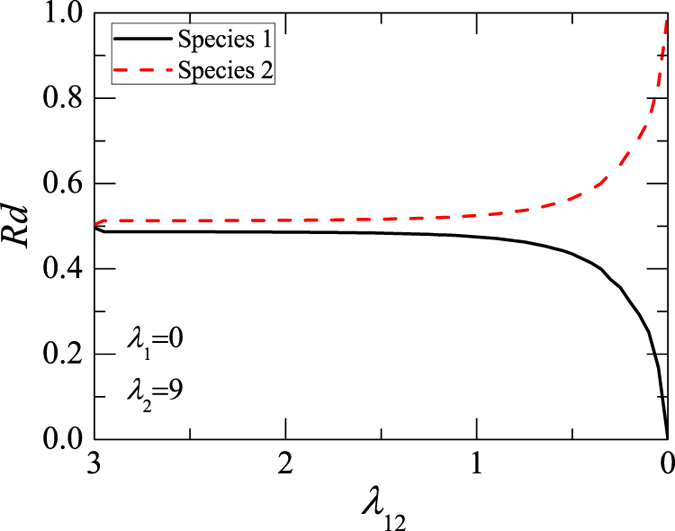
Correspondence relationship between *Rd*_*j*_(*t*) and *λ*_1,2_. Its values refer to the symbiotic DB and are gained from Eq. (12), where *k*_1_ = 40 and *k*_2_ = 42. The solid and dotted lines represent those of species 1 and 2, respectively. Here *λ*_1,1_ = 0 and *λ*_2,2_ = 9.

We systemically calculate numerically the correspondence relationship between the *Rd*_*j*_(*t*) and *λ*_1,2_, shown in Fig. 5. It is obtained in the similar way used in Fig. 4, where *λ*_1,2_ varies a time every 200 time steps. It is worth noting that we had calculated *Rd*_*j*_(*t*) of the DB for different values of *λ*_2_, and the results show that they have no obvious difference. It is obvious that there is a selective distillation phenomenon in the dynamics of a stable symbiotic DB of two-species BECs.

## Methods

To investigate the formation of two-species BECs in an open optical lattice, we supplement the standard DNLSEs with a local dissipation at the two edges of the lattice. They are given by


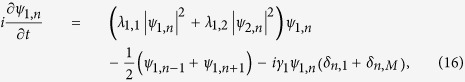



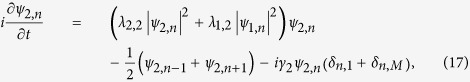


where *δ*_*n*,1_ and *δ*_*n*,*M*_ are delta functions. *γ*_*j*_ describes the atom loss from the boundary of the optical lattices. The optical lattices with leaking edges can be realized experimentally by separating continuous microwave or Raman lasers, where *γ*_*j*_ can be estimated within a mean-field approximation^27^,^58^.

## Conclusion

We have numerically investigated the formation of DBs in two-species BECs by DNLSEs in open optical lattices, and found that there is a selective distillation phenomenon in the mixture of two-species BECs. The coupling of intra- and interspecies interaction can lead to the existence of pure single-species DBs and symbiotic DBs (i.e., the two single-species DBs localized together in the same sites), whose formation can be controlled by varying their interactions in the two-species BECs. In this way, one can selectively distil one species from the mixture of two-species BECs, including the mixture of initial condition selected randomly, that of moving symbiotic DBs, and that of a stable symbiotic DB, and can even control the dominant specie fraction by adjusting the interspecies interaction in optical lattices. Maybe our selective distillation of ultracold atomic gas is useful in quantum information storage and quantum information processing based on multi-species atoms.

## Additional Information

**How to cite this article**: Bai, X.-D. *et al*. Selective distillation phenomenon in two-species Bose-Einstein condensates in open boundary optical lattices. *Sci. Rep*. **5**, 17101; doi: 10.1038/srep17101 (2015).
